# The Genetic and Epigenetic Alterations of Plasmablastic Lymphoma: A Narrative Review

**DOI:** 10.3390/cancers17121914

**Published:** 2025-06-09

**Authors:** Michele Bibas, Andrea Antinori, Valentina Mazzotta, Teresa Marafioti, Jorge J. Castillo

**Affiliations:** 1 Department of Clinical Research: Hematology, National Institute for Infectious Diseases “Lazzaro Spallanzani” I.R.C.S.S., 00149 Rome, Italy; 2 Department of Clinical Research, National Institute for Infectious Diseases “Lazzaro Spallanzani” I.R.C.S.S., 00149 Rome, Italy; 3 Department of Clinical Research: Infectious Diseases, National Institute for Infectious Diseases “Lazzaro Spallanzani” I.R.C.S.S., 00149 Rome, Italy; valentina.mazzotta@inmi.it; 4 Department of Cellular Pathology, University College London Hospitals NHS Foundation Trust, London NW1 2PG, UK; 5 Department Research Pathology, Cancer Institute, University College London, London WC1E 6BT, UK; 6 Center for Hematologic Oncology, Dana-Farber Cancer Institute, Boston, MA 02215, USA; jorgej_castillo@dfci.harvard.edu; 7 Department of Medicine, Harvard Medical School, Boston, MA 02114, USA

**Keywords:** plasmablastic lymphoma, genetics, epigenetics, RAS-RAF, JAK-STAT, MCL1, IRF4, NOTCH, integrated multi-omics, DNA sequencing, Copy Number Variations, miRNA profiling, single-cell omics, spatial omics, ATAC-seq

## Abstract

Plasmablastic lymphoma (PBL) is a rare, aggressive B-cell malignancy that often arises in immunocompromised patients (e.g., HIV+) and is frequently associated with Epstein–Barr virus (EBV) infection. PBL shows terminal B-cell/plasma-cell differentiation (loss of CD20/PAX5 and strong expression of plasma-cell markers such as IRF4/MUM1, CD38, and CD138), and about half of the cases harbor MYC translocations. Despite advances in our understanding of this disease, the prognosis remains poor, resulting in a low overall survival rate. PBL shows a complex molecular profile across multiple omics. Several studies report MYC rearrangements in 46–87% of cases. DNA sequencing and targeted panels identify recurrent mutations in the JAK-STAT (25–55%), RAS-RAF/MAPK (21–49%), and NOTCH (19–45%) pathways. DNA methylation signatures and distinct microRNA patterns distinguish PBL, diffuse large B-cell lymphoma and extramedullary plasmacytoma. Epstein–Barr virus status and TP53 alterations further subdivide cases, with Epstein–Barr virus-negative, MYC-positive tumors linked to poorer survival. Single-cell and spatial transcriptomics reveal a relatively uniform tumor microenvironment, highlighting potential targets, such as IRF4 amplification. These findings suggest that integrated multi-omics approaches can improve diagnostic accuracy, refine prognostic stratification, and inform potential targeted therapy strategies for PBL.

## Simple Summary

Plasmablastic lymphoma (PBL) is a rare and aggressive blood cancer primarily affecting people with weakened immune systems, such as those living with HIV. It is often linked to Epstein-Barr virus infection, which can contribute to cancer development. PBL cells resemble plasma cells but lose key markers. Changes in a gene called MYC drive rapid tumor growth. Despite research advances, PBL has a poor prognosis and treatment is challenging. Recent studies have found genetic mutations in pathways controlling cell growth and survival, and PBL has a unique molecular and epigenetic signature. New technologies, such as single-cell and spatial analysis, are helping researchers understand tumor interactions with their environment, potentially leading to more accurate diagnoses and targeted treatments.

## 1. Background of Plasmablastic Lymphoma (PBL)

**Figure 1:**
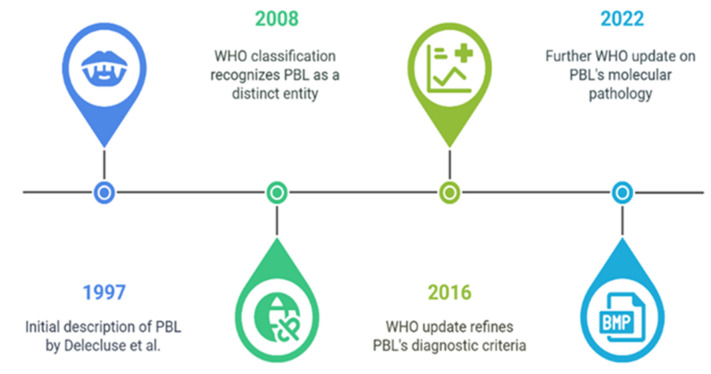
Evolution and recognition of PBL [3].

PBL is a rare and aggressive B-cell lymphoma subtype characterized by plasmacytic differentiation, CD20-negativity, and the expression of plasma cell markers such as CD138 and CD38 [1,2]. Initially described by Delecluse et al. [3] in 1997 as an HIV-associated lymphoma predominantly occurring in the oral cavity, PBL has since been subject to substantial reevaluation regarding its classification and clinical recognition (Figure 1). Early misclassifications as diffuse large B-cell lymphoma (DLBCL) or multiple myeloma (MM) arose due to overlapping morphological and immunophenotypic characteristics [1,2,3,4]. By the 2008 WHO classification, PBL was officially acknowledged as a distinct entity, emphasizing its unique immunophenotypic and genetic profile, with subsequent WHO updates in 2016 and 2022 further refining its diagnostic criteria and integrating advancements in molecular pathology [5,6].

Principal findings indicate that the incidence of PBL has increased worldwide in recent decades, primarily due to enhanced diagnostic detection, especially in HIV-negative and non-immunocompromised patients. The review of PBL in HIV-positive persons concurrently emphasizes the influence of combination antiretroviral therapy (cART/HAART), which has enhanced survival in people living with HIV (PLWH). ART improves general health in suppressed HIV patients by restoring immune function, reducing inflammation, preventing infections, and lowering the risk of HIV-related complications, but has not eradicated the incidence of PBL in regions with a high HIV burden [1,2]. Diagnostic improvements incorporating plasmacytic and molecular indicators, such as CD138, MUM1, and MYC rearrangements, played a role in distinguishing PBL from other lymphomas or plasma cell neoplasms. Nonetheless, obstacles persist, especially in areas with limited resources where diagnostic capabilities and healthcare accessibility are inadequate, limiting an accurate worldwide assessment of the PBL burden [1,2]. This paper aims to elucidate the genetic and epigenetic modifications of PBL for a clinical audience that may lack familiarity with contemporary precision oncology methodologies. Precision oncology methodologies have transformed our understanding and treatment of diverse cancers by focusing on the specific genetic and epigenetic alterations that facilitate carcinogenesis. By tailoring medications to address these modifications, physicians can enhance patient outcomes and reduce side effects.

## 2. Demographic Distribution

The median age at diagnosis varies considerably based on HIV status. HIV-positive individuals are diagnosed at a younger median age (30–40 years) than their HIV-negative counterparts (50–60 years) [1,2]. Pediatric cases are exceptionally uncommon. A male predominance exists in both HIV-positive and HIV-negative groups, with males accounting for 75–80% of cases in the majority of studies. Areas with elevated HIV prevalence, like Sub-Saharan Africa and some regions of Latin America, have a concentration of cases among non-Caucasian populations [1,2].

## 3. Impact of HIV Status

**Figure 2:**
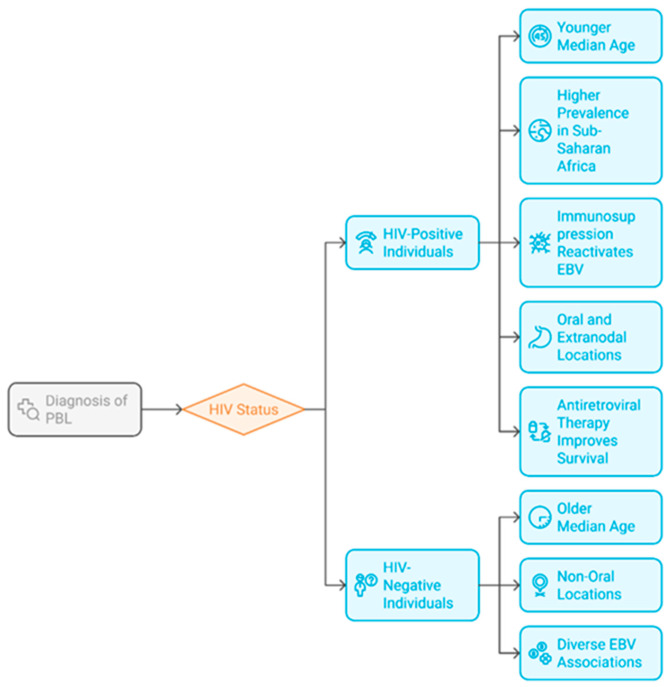
Demographic and clinical characteristics of PBL based on HIV status.

HIV-positive individuals account for nearly 70% of documented occurrences of PBL [1,2,3,4,5,6] (Figure 2) HIV-induced immunosuppression promotes the reactivation of Epstein–Barr virus (EBV), which is observed in approximately 60–75% of PBL cases and is crucial in oncogenesis [4,5,6,7,8]. Antiretroviral therapy (ART) has significantly improved survival rates in these patients, reducing systemic immunosuppression, T-cell exhaustion, chronic inflammation, and the reactivation of EBV. However, the median survival remains suboptimal, ranging from approximately 8 to 18 months [1,2]. The most prevalent primary locations are the oral cavity and extranodal regions, including the gastrointestinal tract. Increased recognition of PBL in HIV-negative persons has been connected with progress in diagnostic standards [1,2]. Compared to HIV-positive patients, these patients receive their diagnosis at an older age, display non-oral primary sites, and have diverse EBV associations. The prognosis is similarly unfavorable, underscoring the aggressive characteristics of PBL regardless of HIV status [1,2].

## 4. Global Trends and Disparity in Data Availability

**Figure 3:**
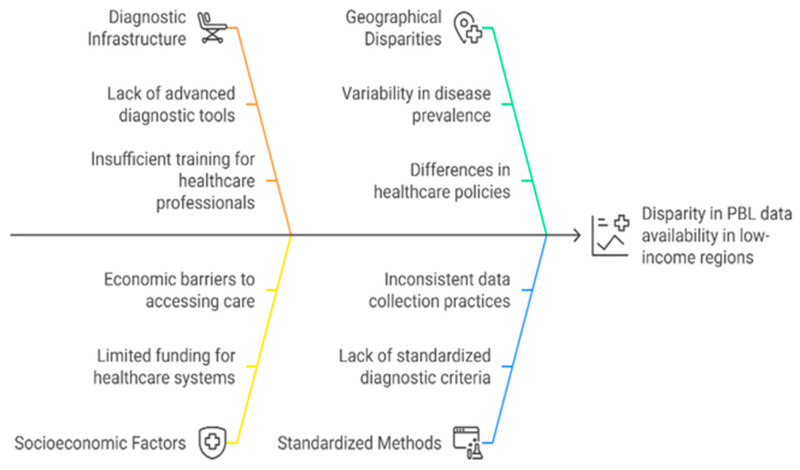
Disparity in PBL data availability.

A significant disparity exists in data availability between high-income and low-income regions (Figure 3). In Sub-Saharan Africa, where HIV prevalence is among the highest worldwide, PBL represents a substantial proportion of lymphomas in HIV-positive populations; however, it is frequently underdiagnosed due to inadequate diagnostic infrastructure [8,9,10,11,12,13,14,15,16]. Latin American studies also underscore regional epidemiological disparities characterized by younger median ages and a higher male preponderance, highlighting the influence of geographical and socioeconomic factors on the presentation and outcomes of PBL. The literature emphasizes the importance of standardized diagnostic methods to enhance the epidemiological collection of data in these regions [8,9,10,11,12,13,14,15,16].

## 5. Incidence

**Figure 4:**
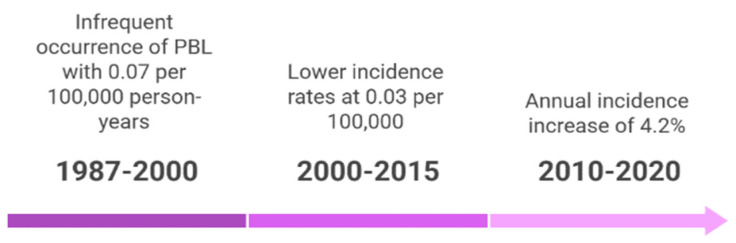
Epidemiological trends of PBL: A timeline.

The occurrence of PBL from 1987 to 2000 is infrequent, with an age-adjusted incidence rate of roughly 0.07 per 100,000 person-years (Figure 4). Estimates derived from data spanning 2000 to 2015 indicated lower incidence rates of 0.03 per 100,000. Extensive research from the SEER and NCDB databases in the U.S. provides significant insights into the epidemiological trends of PBL. However, a recent SEER data study from 2010 to 2020 revealed an annual incidence increase of 4.2%, suggesting a spike in recognition rather than an actual epidemiological explosion [12,13,14].

## 6. Historical Progression and Advancements in Understanding the Genetic and Epigenetic Mechanisms of PBL

**Figure 5:**
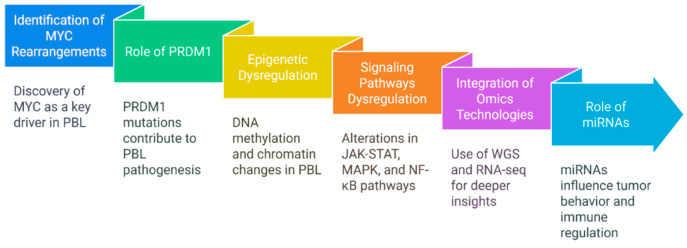
Evolution of PBL pathogenesis understanding.

PBL is distinguished from other B-cell malignancies by its immunophenotype, marked by the absence or low expression of CD20 and the presence of plasma cell markers including CD138, MUM1/IRF4, and cytoplasmic immunoglobulins. It also illustrates specific genetic and epigenetic alterations that contribute to its development. Over the past three decades (Figure 5), research has evolved from cytogenetics to integrated genomic and transcriptome approaches, improving our understanding of cancer progression, immune evasion, and resistance mechanisms.

In the 1990s, the identification of MYC rearrangements, particularly IGH/MYC translocations, in around 50–60% of PBL cases [1,2,17,18,19,20] established MYC as a primary oncogenic driver. Techniques such as FISH and PCR were essential for detection [20]. PBL rarely exhibits BCL2 or BCL6 rearrangements, unlike Burkitt lymphoma, thereby affirming its designation as a separate entity.

In the early 2000s, PRDM1 (Blimp-1), an essential transcription factor for plasma cell development, was recognized as a common change in PBL, particularly in EBV-negative cases [1,2,17]. Loss-of-function mutations or deletions in PRDM1 often correlate with MYC overexpression, intensifying the aggressive phenotype [17,18,19,20,21]. The results demonstrated that MYC alone could not explain the intricacy of PBL.

Since 2010, research has progressively focused on epigenetic dysregulation. Promoter hypermethylation of tumor suppressor genes, including CDKN2A, was frequently seen, impairing cell cycle regulation [21,22,23,24,25]. Mutations in chromatin modifiers, such as EZH2 and KMT2, were identified. EZH2-mediated H3K27 trimethylation suppresses the expression of tumor suppressor genes, thereby facilitating proliferation and immune evasion [25]. These alterations underscored the importance of epigenetics in PBL.

Sequencing revealed common alterations in key signaling pathways, including JAK-STAT, MAPK, and NF-κB [18,22]. STAT3 mutations were more common in EBV-positive patients [8,18], promoting inflammatory and anti-apoptotic microenvironments. IL-6 and IL-10 were likewise linked to the enhancement of JAK-STAT signaling [21]. The overexpression of PD-L1 in EBV-positive PBL facilitates immune evasion, whereas T-cell depletion, macrophage polarization, and activation of the PI3K/mTOR pathway additionally exacerbate disease pathogenesis [18,19,20,21,22,23,24,25].

The advent of whole-genome sequencing (WGS), RNA sequencing (RNA-seq), and single-cell omics has enabled the detection of subclonal changes associated with disease development and therapy resistance, thereby improving the molecular understanding of PBL.

Recent research has highlighted the importance of noncoding RNAs, particularly microRNAs (miRNAs), in tumor control. Distinct miRNA expression profiles were associated with EBV and HIV subtypes [25,26,27]. EBV-derived miR-BARTs and cellular miRNAs, including miR-150-5p, are linked to immunological regulation and aggressive behavior, underscoring the regulatory complexity involved with miRNAs in PBL [25,26,27].

## 7. Multi-Omic Profiling in PBL

Due to the phenotypic overlap between PBL, DLBCL, and MM, high-throughput molecular profiling, including genomic, transcriptomic, and proteomic analyses, has been employed to elucidate their biology, identify biomarkers, and propose targeted therapeutics. Recent studies have integrated next-generation sequencing (NGS) with other omics data to delineate the distinctive molecular landscape of PBL [21,22,23].

### 7.1. Genomic Modifications (DNA Sequencing and Copy Number Variations)

High-throughput DNA investigations in PBL have utilized whole-exome sequencing (WES), targeted gene panels, SNP microarrays, and copy-number arrays on tumor tissue, commonly Formalin-Fixed Paraffin-Embedded (FFPE) [4,5,6,7,8,9,10,11,12,13,14,15,16,17,18,19,20,21,22,23,24,25,26,27,28]. Frontzek et al. [28] conducted whole-exome sequencing and genome-wide copy-number profiling on 96 cases of PBL, demonstrating that PBL possesses unique genetic characteristics compared to DLBCL or myeloma [28]. Recurrent somatic mutations influence oncogenic signaling pathways and tumor suppressor genes. Frequently mutated genes involve parts of the RAS/MAPK and JAK/STAT pathways (e.g., NRAS, KRAS, BRAF, STAT3, OSMR, PIM1, SOCS1), in addition to TP53 and chromatin regulators (TET2, KMT2A/D) [21,28]. Integrated analysis revealed widespread disruption of IL-6/JAK–STAT signaling, with WES/RNA-seq data indicating that around 64% of patients exhibited abnormalities (mutations or copy-number variations) in this pathway [17]. Other studies identified distinct trends, such as the enrichment of STAT3 mutations in HIV-positive individuals (47% compared to 10% in HIV-negative cases, *p* = 0.003) [19]. A separate study identified recurrent inactivating mutations in PRDM1 (BLIMP1) and SOCS1 [20]. Additionally, substantial variations in copy numbers are indicative. There were frequent focal amplifications of chromosome 6p (which encompasses IRF4/MUM1) and 1q (which contains MCL1) [28]. For instance, IRF4 was elevated locally in approximately 30% of instances. Approximately 32% of samples exhibit additional recurrent increases in 8q24.13 (TRIB1) [28,29]. The CNA profile of PBL is intermediate between MM and DLBCL [30,31]. Around 45–50% of PBL cases contain MYC translocations, which are frequently associated with the IgH locus. These translocations can be detected using FISH or PCR techniques [28,29,30,31].

### 7.2. Transcriptomic and miRNA Profiling

RNA-based omics, including mRNA and miRNA profiling, have uncovered a distinct expression profile in PBL. Bulk RNA sequencing of PBL samples demonstrated an increased activity of immune and oncogenic pathways; specifically, IL-6/JAK–STAT3 signaling, NF-κB, and MYC target genes were markedly enriched, particularly in EBV-positive cases [21,32]. Witte et al. [22] identified elevated IL-6/JAK–STAT3 and NF-κB signatures by RNA-seq, along with significant transcriptional upregulation of PI3K/AKT/mTOR in EBV+ PBL. Comparative transcriptomics has proven to be a valuable tool. Nano String expression profiling utilizing a 770-gene panel has shown that PBL clusters distinctly from activated B-cell DLBCL (ABC-DLBCL). Gene set enrichment analysis identified the RAS pathway as the most significantly over-represented signature in PBL [32]. Multiple RAS/MAPK genes (NRAS, RAF1, SOS1, SHC1) exhibited considerable upregulation in PBL compared to ABC-DLBCL, alongside components of the Wnt/β-catenin pathway (FZD3, FZD7, WNT5A/B, WNT10B, c-MYC) [32]. PBL also exhibited overexpression of Wnt inhibitors (DKK1, SFRP2), indicating intricate pathway regulation. The results reveal specific signaling dependencies (e.g., RAS and Wnt) that may be therapeutically targeted [32]. Small RNA sequencing (miRNA profiling) considerably elucidated PBL taxonomy. A study established a miRNA signature specific to PBL that distinctly differentiated PBL from its closest mimics (Burkitt lymphoma and plasmacytoma) and indicated two subgroups of PBL: one with Burkitt-like miRNA profiles and the other with plasmacytoma-like profiles [26,32]. This heterogeneity was associated with clinical characteristics (HIV status, MYC status, etc.). Recent integrative RNA and miRNA analyses revealed that PBL with MYC translocation had a unique microRNA profile, particularly characterized by the downregulation of miR-150-5p [27]. The depletion of miR-150 resulted in the overexpression of E2F3 and survivin (BIRC5), facilitating cell-cycle progression. Functional experiments demonstrated that the reintroduction of miR-150 or the pharmacological inhibition of E2F3 and survivin resulted in G1 arrest and cellular apoptosis in PBL models. Consequently, transcriptome profiling (mRNA and miRNA) has delineated PBL-specific signatures and identified potential therapeutic targets (e.g., the miR-150/E2F3/survivin axis) [27,32].

### 7.3. Precision Oncology via Multi-Omic Integration

A significant advancement has been the combined use of DNA sequences and transcriptomics, along with proteomic and immunohistochemical data, to categorize PBL and drive treatment strategies [32,33,34,35]. In the clinical area, multi-omic profiling has facilitated decision-making in PBL using “molecular tumor board” approaches. The incorporation of Molecular Tumor Boards into diagnostic protocols is endorsed by global guidelines, including those from ESMO and NCCN, which highlight their significance for personalized and evidence-based oncology treatment [33]. A recent proof-of-concept study including 14 primary-refractory PBL patients utilized a comprehensive methodology. Each patient’s biopsy underwent whole-exome sequencing (WES), whole-transcriptome sequencing, and targeted immunohistochemistry (IHC) for CD19, CD30, CD38, and CD79B [34]. This integrated analysis consistently identified at least one actionable target (e.g., tyrosine kinases, JAK/STAT system, cell-cycle genes, immune evasion markers, B cell markers) with sufficient evidence to suggest a therapeutic approach. All 14 cases exhibited either a druggable mutation or an IHC marker, such as CD38 or CD30, that might be targeted [34]. Treatment candidates were prioritized according to evidence (NCT/DKTK and ESCAT criteria); numerous patients might have been administered JAK inhibitors, PI3K/MTOR inhibitors, BCL2 inhibitors, immune checkpoint inhibitors, or antibody-drug conjugates based on their omics profile. This work demonstrates that, even for a highly aggressive lymphoma, multi-omic data can provide personalized therapy recommendations. This personalized approach enhances the likelihood of treatment success and minimizes unnecessary side effects by focusing on therapies most likely to be effective for each patient [34]. Ultimately, the integration of multi-omic data into clinical decision-making represents a significant advancement in the management of aggressive lymphomas [31,32,33,34].

### 7.4. Single-Cell and Spatial Omics

Single-cell and spatial omics methodologies, such as single-cell RNA sequencing (scRNA-seq) and spatial transcriptomics, are potent instruments for elucidating cellular heterogeneity and microenvironmental interactions in PBL. These approaches may yield comprehensive insights into the varied cell types within the lymphoma and their spatial arrangement, elucidating their interactions with the surrounding microenvironment [35,36]. Recently, an in situ single-cell transcriptomic analysis conducted by the Lymphoma/Leukemia Molecular Profiling Project (LLMPP) revealed a sparse tumor microenvironment (TME) in PBL, with the predominant non-malignant cell types comprising, on average, 10% macrophages, 7% NK/T cells, and 4% stromal cells among all identified cell types [35]. EBV+/MYC+ cancers had a greater immune cell population, characterized by elevated macrophage levels (median 12%) and NK/T cells (median 9%). At the same time, EBV-/MYC tumors contained the lowest levels of immune cells (median 4% macrophages and 1% NK/T cells). Regarding malignant cell phenotypes, they identified a distinctive group of SPP1-expressing cells that were prevalent in EBV+/MYC+ tumors, as well as a population of CD44-expressing cells that were predominant in EBV+/MYC- tumors [35]. Other highly relevant studies to this goal have focused on closely related entities such as EBV+ DLBCL and primary CNS lymphoma (PCNSL) (single-cell and spatial characterization of plasmablast-like lymphoma cells in primary central nervous system lymphoma) [36].

### 7.5. ATAC-Seq and Chromatin Accessibility in PBL

The Assay for Transposase-Accessible Chromatin utilizing sequencing (ATAC-seq) is a high-throughput methodology that identifies open chromatin regions, providing insights into transcriptional control and epigenomic structure. In PBL, ATAC-seq may serve as a valuable instrument to elucidate the epigenetic dysregulation that contributes to disease pathogenesis. Initial investigations using ATAC-seq in PBL have revealed distinct chromatin accessibility patterns that differentiate it from analogous conditions, such as MM and DLBCL [29,30,31,32,33,34]. These profiles frequently correspond with the activation of oncogenic transcription factors (e.g., MYC, IRF4) and underscore the anomalous epigenetic environments influenced by intrinsic mutations (e.g., in chromatin modifiers such as EP300, TET2) and extrinsic factors, including EBV infection. Specifically, EBV-positive PBL patients may have unique epigenetic patterns characterized by immune evasion markers [29,30,31,32,33,34].

### 7.6. Exosomes and microRNAs in PBL Pathogenesis

Recent studies suggest that exosomes and miRNAs may be very important in the cause and progression of PBL, especially in immunocompromised people. Malignant cells release membrane-bound extracellular vesicles called exosomes, which are becoming more and more acknowledged as ways for cells to talk to one another in the tumor microenvironment [35]. Researchers have found that exosomes in aggressive B-cell lymphomas and plasma cell-derived neoplasms convey oncogenic proteins (including MYC), immunomodulatory chemicals, and miRNAs that help blood vessels grow, make stroma more flexible, and help the cancer cells avoid the immune system [36].

These things help tumors grow and may help them spread to other parts of the body. There is not much research focused on PBL-derived exosomes, but when compared to diffuse large B-cell lymphoma (DLBCL) and multiple myeloma, they seem to have a comparable role in causing cancer [36].

Concurrently, miRNA dysregulation has been associated with PBL, with specific miRNAs (such as miR-21 and miR-155) recognized for promoting B cell proliferation and inhibiting apoptosis in lymphoproliferative disorders. Significantly, miR-150-5p, regarded as a tumor suppressor that targets the MYB and NOTCH pathways, has been observed to exhibit markedly reduced activity in individuals with lymphoma who are HIV-positive. This indicates that it may contribute to the aggressive nature of HIV-associated PBL [37].

Moreover, in cases of Epstein–Barr virus (EBV)-positive primary B-cell lymphoma (PBL), EBV-encoded microRNAs, especially those belonging to the BART family, may facilitate immune evasion and the maintenance of latent viral persistence. The concept that PBL may utilize exosome-mediated transfer of oncogenic miRNAs to alter the tumor microenvironment and evade immune surveillance is biologically feasible and necessitates additional exploration via high-throughput characterization of exosomal content in PBL patients [38].

## 8. The Role of Genetic Modifications in the Differential Diagnosis of PBL, MM and DLBCL

There are significant challenges associated with the differential diagnosis of PBL, MM, and other aggressive lymphomas, since these tumors exhibit overlapping clinical, morphological, and molecular characteristics (Table 1: clinical differences). Key points of differentiation have concentrated on the prevalence and processes of particular gene mutations (for example, MYC, STAT3, PRDM1, and TP53 in PBL; KRAS, NRAS, BRAF, and TP53 in MM; EZH2, BCL2, and BCL6 in DLBCL), as well as unique structural rearrangements (Table 2: genetic differences and Table 3: epigenetic differences). However, despite the increased interest in the function that epigenetic data plays in molecular subtyping and prognostics, there is still a lack of integration of epigenetic data into diagnostic frameworks.

The following is a summary of the most significant genetic differences:

### 8.1. PBL

a. MYC Rearrangements: MYC rearrangements are the most consistent genetic signature of PBL, since they have been reported in between 45 and 87 percent of patients [18,19,20,21,35,36,37,38,39,40,41,42,43]. The immunoglobulin heavy chain locus (IGH-MYC) is frequently involved in translocations, which suggests that activation-induced cytidine deaminase (AID)-mediated processes are specific to lymphoid malignancies [20]. The distinction between double- or triple-hit DLBCL and PBL is that the latter does not have simultaneous BCL2 or BCL6 rearrangements, which is an essential feature for differential diagnosis [20,28,29,30]. When compared to MM, where MYC translocations are uncommon and AID-chromatin targeting is not detected, the MYC rearrangements that occur in PBL have a significantly higher frequency and a different mechanism of action [36,37,38,39,40,41,42,43].

b. Mutational Landscape: The mutational landscape of the JAK/STAT pathway activation mutations in STAT3, which is a major activator of oncogenic transcription, and aberrations in the JAK/STAT pathway are essential in the pathogenesis of PBL [25,26,27,28,29,30,31,32,33,34,35,36,37,38,39,40,41,42,43]. In the context of NF-κB Pathway Dysregulation, it is common for mutations to develop in PRDM1, which is known as Blimp1, the master regulator of plasma cell differentiation, and CARD11. These mutations have the effect of upregulating survival and proliferation pathways [18,19,20,21,22,23,24,25,26,27,28,29,30,31,33]. Loss of Function of TP53: Between thirty and fifty percent of cases of PBL contain TP53 mutations, which are responsible for genomic instability and aggressive behavior [18,19,26]. RAS Pathway Alterations: KRAS, NRAS, and BRAF mutations are reported in roughly 10-20% of PBL cases, with considerable overlaps in pathway activation with MM [11,12,13,14,15,16,17,18,19,32,33,34,35,36,37,38,39,40,41,42].

c. *Copy Number Alterations (CNAs):* Amplification of Chromosome 3q has been linked to unfavorable prognosis. The gain of 3q frequently results in the amplification of oncogenes such as MYC, which are recognized for promoting cell proliferation and survival. Deletion of Chromosome 17p may result in the inactivation of the tumor suppressor gene TP53 [25,26,27,28,29,30]. The loss of TP53 leads to deficient DNA damage repair, resulting in heightened genomic instability and tumor advancement. Deletion of the CDKN2A/B gene on chromosome 9p, which encodes tumor suppressors such as p16INK4A, is a typical phenomenon in PBL. The absence of these genes results in the impairment of cell cycle regulation and uncontrolled cell proliferation. Chromosome 18 Gain: Certain studies have observed gains in chromosome 18, which may affect areas that regulate cell signaling and survival pathways. Deletion of Chromosome 13q: Alterations in this region may affect the RB1 gene and other genes that regulate the cell cycle and apoptosis, thereby facilitating lymphoma progression [22,23,24,25,26,27,28,29,30,38,39,40,41,42,43,44,45,46,47].

d. *EBV Status:* The presence of EBV association is a distinguishing feature of PBL, particularly in immunocompromised patients, such as those who are on HIV/AIDS treatment. The presence of EBV-encoded RNA in PBL is positive in roughly sixty to seventy-five percent of patients [24,31]. This is determined through the use of EBER in situ hybridization [17,18,31,38]. Research has shown that EBV latency type I/II is involved in the process of driving transcriptional programs that involve the activation of the MYC and JAK-STAT pathways. EBV is helpful in differentiating EBV-positive PBL from EBV-negative plasma cell neoplasms, since MM is persistently negative for EBV [39,40,41,42,43,44,45,46,47].

### 8.2. Multiple Myeloma

MM exhibits a distinct genetic profile to PBL and DLBL. Mutations in KRAS and NRAS are present in approximately 40% and 30% of MM patients, respectively, often coinciding with BRAF mutations at a rate of around 10%, underscoring the significance of the MAPK signaling pathway [18,21]. Mutations in TP53 (about 10–20%) and recurrent chromosomal amplifications (such as gain of 1q) and deletions (such as del17p) are implicated in the pathophysiology of MM. MYC rearrangements are infrequent (~2–5%), mechanistically separate from PBL or DLBCL (e.g., independent of AID pathways), but amplifications or overexpression can still occur in aggressive MM [15]. MM is characterized also, by specific immunoglobulin translocations, including t(11;14) (IGH-CCND1), t(4;14) (IGH-MMSET/NSD2), and t(14;16) (IGH-MAF), which lead to distinctive molecular characteristics [28,32,34,35,36,37,38,39,40]. These translocations are not characteristic of PBL or DLBCL, therefore providing diagnostic specificity [41,42,43,44,45,46,47,48,49].

Hyperdiploidy, characterized by trisomies of odd-numbered chromosomes (e.g., +3, +5, +7, +11), is a defining feature of MM and is nearly absent in PBL and DLBL [34]. MM’s substantial dependence on hyperdiploidy and clonal immunoglobulin rearrangements distinguishes it from PBL both phenotypically and genomically [28,45,46,47,48,49,50,51].

### 8.3. DLBCL

DLBCL, especially high-grade double- or triple-hit variants, has a genomic profile that overlaps with PBL [5,6]. However, distinctions include BCL2 and BCL6 rearrangements, which are involved in regulating apoptosis and cell survival. BCL2 overexpression is associated with an increased risk of lymphoma, whereas BCL6 plays a crucial role in maintaining germinal center B cells [39,40,41,42,43]. MYC translocation: Although it is less common than in PBL, MYC rearrangements are also observed in a subset of LBCL, often in the form of double-hit lymphoma (co-occurring MYC and BCL2/BCL6 translocations), which has a poor prognosis. TP53 mutations: Mutations in TP53 are often associated with an aggressive course and a poor prognosis. CDKN2A alterations: Loss of the CDKN2A tumor suppressor gene is common, particularly in high-grade LBCL [28,51,52,53,54,55,56].

## 9. The Role of Epigenetic Modifications in the Differential Diagnosis of PBL, MM and DLBCL

The following is a summary of the most significant epigenetic differences:

### 9.1. PBL

The role of epigenetic mechanisms, such as promoter DNA methylation, histone modifications, and microRNA (miRNA) dysregulation, in the pathogenesis and progression of this disease remains incompletely defined. A central question is whether alterations in these epigenetic layers lead to transcriptional and phenotypic changes that could be exploited therapeutically.

Early transcriptomic studies demonstrated the downregulation of B-cell receptor signaling and identified unique gene expression signatures in PBL relative to DLBCL [18,22,27,28,56]. Subsequent comprehensive genomic and transcriptomic analyses have delineated the mutational landscape of PBL, uncovering recurrent alterations in signaling pathways such as JAK-STAT, MAPK, and chromatin-modifying genes (e.g., TET2, EP300), as well as distinct patterns of gene expression shaped by EBV status [18,21,49].

Two studies have specifically addressed miRNA regulation in PBL. Ambrosio et al. [26]. performed small RNA sequencing on 23 primary PBL biopsies, identifying biologically distinct disease subgroups based on miRNA expression correlated with clinical features, such as HIV status and MYC rearrangement. However, this work was descriptive, lacking functional validation of miRNA targets [26]. Verdú-Bou et al. [27] identified downregulation of miR-150-5p in PBL samples and demonstrated, using a PBL-1 cell line model, that restoration of miR-150-5p suppresses proliferation and induces apoptosis via repression of E2F3 and BIRC5, thus establishing a mechanistic link between miRNA loss and oncogenic phenotypes. Notably, all functional assays in this study were performed in cell lines rather than primary patient tissues. Promoter hypermethylation in tumor suppressor genes such as PRDM1 (Blimp1) and CDKN2A is reported in PBL, frequently overlapping with EBV-driven epigenetic reprogramming [25,26,57,58]. EBV latent membrane protein 1 (LMP1) interacts with host chromatin remodeling proteins, influencing histone methylation and promoter silencing in PBL [24,25,28,47,48,49,50,51,52,53,54,55,56,57].

### 9.2. Multiple Myeloma

Studies using CRISPR/Cas9, RNA interference, and degrader approaches have confirmed that NSD2 and its reader domains are essential for myeloma cell survival, chromatin targeting, and maintaining oncogenic gene expression. Physical and functional interactions between NSD2 and other chromatin regulators, such as EZH2 and SMARCA2, further reprogram the chromatin landscape, promoting myeloma growth, altering enhancer activity, and sustaining drug resistance [59]. Elevated H3K27me3-mediated silencing of tumor suppressors and B-lineage genes correlates with myeloma aggressiveness and poor prognosis [60]. Inhibition of EZH2 reverses these repressive marks and induces MM cell apoptosis, highlighting the therapeutic potential of targeting histone methylation pathways. Whole-genome DNA methylation analyses reveal global hypomethylation and focal hypermethylation in myeloma, disrupting normal gene regulation and associated with loss of differentiation, disease progression, and subtype-specific methylation signatures [58]. CRISPR-based tools show that targeted methylation of enhancers can directly repress oncogenic gene expression [61].

### 9.3. DLBCL

Genome-wide DNA methylation studies have shown that different subtypes of LBCL have distinct methylation signatures that have prognostic implications [62]. Recurrent somatic mutations in epigenetic regulators, such as TET2, EZH2, KMT2D, and CREBBP/EP300, significantly impact the DLBCL epigenome. TET2-deficient cases exhibit widespread promoter and enhancer hypermethylation, which contributes to transcriptional silencing of immune signaling and differentiation genes [63,64]. These methylation changes are conserved across murine models and human patient samples, and large-scale methylation profiling has shown that increased methylation heterogeneity is associated with advanced disease and poorer survival. Histone modification studies in DLBCL have focused on the consequences of mutations in chromatin-modifying enzymes [65]. Gain-of-function mutations in EZH2 result in increased H3K27 trimethylation at promoters of key differentiation genes, leading to a block in terminal differentiation and maintenance of the germinal center phenotype [66]. Loss-of-function mutations in KMT2D reduce H3K4me3 at enhancers and activate specific signaling pathways [62,63,64,65,66].

## 10. Prognostic Implications of Specific Genetic and Epigenetic Alterations in PBL

**Figure 6:**
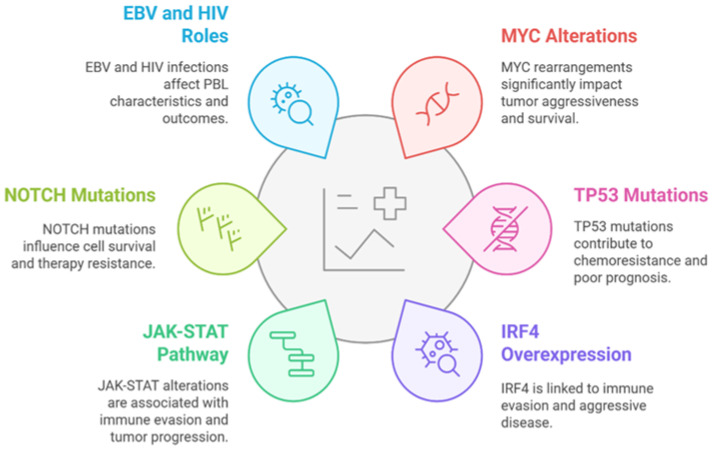
Factors influencing prognosis in PBL.

The high mutational burden in PBL, particularly in EBV-negative cases, and the limited efficacy of standard lymphoma therapeutic regimens, including CHOP and CHOP-like therapies, underscore the urgent need for identifying factors influencing prognosis and response to therapy (Figure 6).

### 10.1. MYC: The Central Driver of Aggressiveness and Prognosis

MYC is among the most commonly modified genes in PBL, with alterations like translocations or amplifications affecting prognosis and therapeutic resistance. Approximately 50–87% of PBL cases have MYC rearrangements, predominantly characterized by t(8;14)(q24;q32) translocations affecting the immunoglobulin heavy chain gene (IGH), resulting in MYC overexpression [1,18,19,47]. These modifications promote fast tumor growth, impede apoptosis, and reconfigure metabolic pathways, including the Warburg effect, to facilitate aggressive proliferation [1,2,47]. MYC rearrangements are consistently related with diminished overall survival (OS) and progression-free survival (PFS), with research indicating adverse outcomes in both HIV-positive and HIV-negative individuals [1,2,26,47]. MYC rearrangements independently resulted in considerably poorer survival (OS: HR 2.071, *p* = 0.005) [45]. Nonetheless, differences are present: in a study with daratumumab-based therapy, no significant association was identified between MYC level and treatment response, indicating that MYC’s influence may differ based on the regimen [44,45]. EBV-positive patients typically exhibit intact MYC due to alternative oncogenic factors such as JAK-STAT activation, whereas myeloma-like MYC-driven aggressive metabolism often prevails in EBV-negative PBL [44,45].

### 10.2. TP53 Mutations: A Contributor to Resistance

TP53 mutations or deletions are present in roughly 20–40% of PBL cases and are more prevalent in EBV-negative subgroups, where they are significantly linked to worse prognosis [19]. These mutations impair apoptotic processes, exacerbating the chemoresistance attributed to MYC, especially when both changes are present together [19]. HIV-negative PBL patients exhibit elevated TP53 mutation frequencies, indicating unique carcinogenic pathways in immunocompetent individuals [1,2,21,45].

### 10.3. IRF4 (MUM1): A Marker of Differentiation and Immune Modulation

IRF4/MUM1, a plasma cell marker that is overexpressed in almost all PBL cases, is crucial in immune evasion and carcinogenesis [39]. IRF4 is recognized for regulating transcriptional programs that facilitate plasma-cell development; nevertheless, its function in PBL-specific survival or chemoresistance remains ambiguous [21,26]. IRF4 mutations are infrequent but are linked to more aggressive disease phenotypes when they occur [44].

### 10.4. JAK-STAT Pathway: A Probable Therapeutic Target in Tumor Immunomodulation

The JAK-STAT system, specifically STAT3, is among the most commonly altered signaling pathways in PBL, with mutations identified in 20–42% of patients [18,19,48]. These alterations stimulate downstream effectors, including PD-L1, facilitating immune evasion and tumor advancement. Mutations in the SH2 domain of STAT3, primarily observed in EBV-positive cases, augment phospho-STAT3 activity, which is associated with greater aggressiveness and worse survival rates [18,48].

### 10.5. NOTCH Pathway: A Driver of Survival and Therapy Resistance

Mutations in NOTCH1 and NOTCH2, reported in 15–20% of PBL cases, influence cell survival, apoptosis, and chemoresistance [18,21]. NOTCH signaling is essential for lymphocyte development; yet, its abnormal activation in PBL promotes proliferation and immune evasion, connecting with NF-kB and canonical signaling pathways [18].

## 11. Differences Between EBV-Positive and EBV-Negative PBL

EBV infection is detected in approximately 75% of PBL cases, particularly in HIV-positive individuals, where it contributes to oncogenesis through its latent proteins, including EBNA1, LMP1, and LMP2A [24,39,46,67]. EBV-positive cases often exhibit mutations in the JAK-STAT pathway (particularly STAT3), NOTCH1, alongside a significant occurrence of MYC rearrangements.

These patients frequently have a reduced number of mutations in epigenetic regulators, such as TET2 and KMT2D, compared to EBV-negative cases [18,48,67] (Table 4).

Laurent et al. provide evidence that immune checkpoint pathways, including PD-L1, are upregulated in EBV+ PBL, though the most prominent expression is on microenvironmental (not necessarily neoplastic) cells; explicit tumor cell PD-L1 positivity is relatively less common [68].

The PBL tumor microenvironment (TME) is generally sparse. Recent analyses of EBV⁺ vs. EBV⁻ PBL highlight significant differences: EBV⁺ PBL tend to have more immune infiltration but also immune evasion, whereas EBV⁻ PBL appear more “immune-cold EBV⁺. PBLs exhibit abundant leukocyte infiltrates and T-cell activation signatures, yet express high levels of PD-L1 and other checkpoint markers.

In these EBV⁺ cases, PDL1 is often upregulated on tumor cells, and CD8⁺ T-cells show exhaustion markers. By contrast, EBV⁻ PBLs tend to display PD-L1 on tumor-associated macrophages rather than on malignant cells, and have fewer CD8⁺ T-cells. Thus, PBLs exploit checkpoints (PD-1/PD-L1, TIM-3) to evade immunity.

The pronounced differences by EBV status imply that future single-cell/spatial studies should stratify by EBV. Detailed spatial maps could show, for example, whether CD163⁺ macrophages and CD8⁺PD-1⁺ T cells localize to distinct niches around the tumor [25,68].

EBV-positive PBL is more prevalent among HIV-positive individuals, younger males, and frequently manifests in the oral cavity. These cases typically have a more favorable response to chemotherapy and extended survival [2,48,68].

EBV-negative PBL is more prone to contain more mutations in epigenetic modifiers (TET2, KMT2D), TP53, and genes associated with the RAS pathway (KRAS, NRAS). MYC rearrangements are present, although less frequently than in EBV-positive patients.

EBV-negative PBL occurs more commonly in older, HIV-negative, or immunocompetent individuals, with increased extraoral manifestations and a usually poorer prognosis [24,28,32,69,70,71,72].

HIV-positive patients often present with advanced-stage disease, but intriguingly, HIV status itself has inconsistent effects on prognosis [1,2,55]. HIV co-infection contributes to PBL through systemic immunosuppression, T-cell exhaustion, and chronic inflammation. The interplay of HIV and EBV remains critical to understanding variability in survival outcomes [55,72].

However, the role of dual HIV/EBV infection in determining survival outcomes remains underexplored, with most studies either analyzing these factors separately or failing to stratify outcomes by co-infection [20,32,55,69,70,71,72].

**Table 4:** Comparison of EBV-positive and EBV-negative PBL.

Feature	EBV-Positive PBL	EBV-Negative PBL
Prevalence in PBL	Approximately 75% of cases	Less common
Common in HIV+ individuals	Yes	Less common, more in HIV-negative
Demographic Trends	Younger males, often HIV-positive	Older, immunocompetent individuals
Site of Involvement	Oral cavity predominant	More extraoral involvement
Oncogenic EBV Proteins	EBNA1, LMP1, LMP2A	Not applicable
Common Genetic Alterations	STAT3, NOTCH1	Frequent in TP53, KRAS, NRAS
MYC Rearrangement Frequency	High	Lower
Mutations in Epigenetic Regulators	Fewer (e.g., TET2, KMT2D)	More frequent (e.g., TET2, KMT2D)
Mutations in Other Genes	Less frequent TP53/RAS pathway mutations.	Common TP53, RAS pathway mut.
Immune Microenvironment	More immune infiltrates, sparse TME	“Immune-cold”, fewer infiltrates
PD-L1 Expression	Primarily on microenvironment cells; tumor cell expression in some	Primarily on tumor-associated macrophages
CD8+ T-cell Infiltration	Abundant, exhaustion markers (PD-1+, TIM-3+)	Fewer CD8+ T-cells
Immune Evasion Mechanisms	PD-1/PD-L1, TIM-3 checkpoints exploited	Less prominent checkpoint activation
Prognosis	More favorable	Poorer
Response to Therapy	Better response to chemotherapy, extended survival	Generally poorer outcomes
Clinical Recommendations	Stratify studies by EBV; consider spatial immune profiling	Requires distinct immunogenomic characterization

## 12. Therapeutic Strategies Guided by Genetic and Molecular Insights

Despite its aggressive nature, treatment of PBL often relies on therapeutic regimens borrowed from other hematological malignancies, such as DLBCL or MM. Standard chemotherapies, including CHOP (cyclophosphamide, doxorubicin, vincristine, prednisone) or more intensive regimens such as EPOCH (etoposide, prednisone, vincristine, cyclophosphamide, doxorubicin), have shown limited success, with high rates of relapse and poor overall survival ranging from 6 to 15 months [1,2].

**Figure 7:**
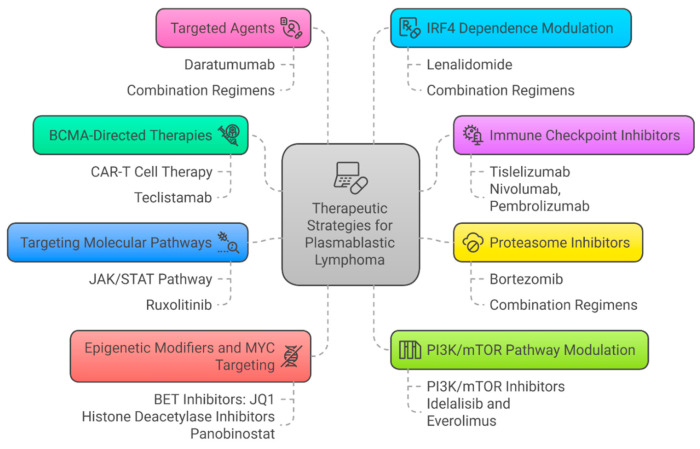
Therapeutic strategies guided by genetic and molecular insights.

While it is true that CHOP and EPOCH may not target specific aspects of plasma cell biology, MYC-driven oncogenesis, epigenetic dysregulation, and immune evasion mechanisms, they have still shown efficacy in treating some patients with PBL. In recent years, advances in molecular characterization of PBL have identified potential actionable drivers (Figure 7), sparking interest in personalized or targeted therapies informed by genetic findings [34,73,74].

**BCMA-Directed Therapies** (preclinical, clinical): B-cell maturation antigen (BCMA), a transmembrane glycoprotein crucial to plasma cell development, is significantly expressed in PBL, mirroring its expression in MM [75]. Its significant presence in PBL indicates it may function as a highly selective target for immunotherapy. A retrospective research study demonstrated uniform BCMA expression in all analyzed PBL samples, thereby reinforcing therapeutic potential [75]. A clinical example demonstrated the effective use of BCMA-directed CAR-T cell therapy in a refractory PBL patient, achieving complete remission for over a year, with no major side effects [76]. Teclistamab, a bispecific T-cell engager targeting BCMA, exhibited therapeutic responses in two relapsed/refractory cases, validating BCMA as an attractive target in PBL patients [77].

**Immune Checkpoint Inhibitors (ICIs)** (preclinical, clinical): Activation of immune checkpoints, specifically the PD-1/PD-L1 axis, is noted in certain subsets of PBL, including those associated with EBV and HIV. Elevated PD-L1 expression is associated with immunological exhaustion and represents a possible biomarker for immune checkpoint inhibitor utilization [1,2,25,68]. Here is some clinical evidence of the efficacy of immune checkpoint inhibitors (ICIs): A PD-L1-positive PBL patient attained remission after receiving nivolumab therapy and subsequent allogeneic stem cell transplantation [74]. A patient with recurrent EBV-positive, HIV-negative PBL treated with pembrolizumab and radiation achieved durable remission [77]. A refractory PBL case achieved complete remission following treatment with the PD-1 inhibitor tislelizumab in conjunction with lenalidomide, underscoring the possibility of combining immune checkpoint inhibitors with immunomodulatory drugs [78].

**Proteasome Inhibitors** (preclinical, clinical): Due to the similarities in molecular and clinical characteristics between PBL and plasma cell diseases, proteasome inhibitors such as bortezomib have been investigated for the treatment of PBL. Retrospective studies have shown substantial enhancements in response rates and survival when bortezomib is incorporated into combination regimens such as dose-adjusted EPOCH [75,76,77,78]. A specific case of prolonged remission was attained with the combination of bortezomib, autologous stem cell transplantation (ASCT), and lenalidomide maintenance [79,80,81,82,83].

**Targeting molecular pathways vulnerabilities** (functional genomic, preclinical): Ruxolitinib has been proposed to target constitutive activation of the JAK/STAT pathway in PBL cases with STAT3 or JAK1 mutations, with preclinical evidence supporting its effectiveness [18,19,34,73].

**Epigenetic Modifiers and MYC Targeting**(functional genomic, preclinical): Despite the challenges of directly targeting MYC, epigenetic drugs such as BET inhibitors (e.g., JQ1) and histone deacetylase inhibitors (e.g., panobinostat) offer an indirect route, suppressing MYC-driven programs [34,73]. These strategies remain preclinical but represent promising avenues for future exploration.

**PI3K/mTOR Pathway Modulation**(functional genomic, preclinical): The PI3K/mTOR pathway is implicated in the pathogenesis of PBL through mechanisms involving MYC overexpression, EBV-associated signaling, and mTOR activation, but while theoretical therapeutic strategies have been proposed (e.g., PI3K/mTOR inhibitors like idelalisib and everolimus), no clinical trials specifically validating these approaches in PBL have been conducted [34,73].

**Targeted Agents as Daratumumab (anti-CD38) (clinical)**: Mechanistically, daratumumab exerts pleiotropic effects through tumor cell apoptosis, antibody-dependent cellular cytotoxicity (ADCC), complement-dependent cytotoxicity (CDC), antibody-dependent cellular phagocytosis (ADCP), and immune modulation. Efforts to optimize daratumumab efficacy in PBL have increasingly focused on rational combination regimens [34,73,84].

**IRF4 Dependence Modulation (Clinical):** Lenalidomide, which downregulates IRF4, has shown preclinical and clinical efficacy. Cases of refractory PBL treated with lenalidomide as a single agent or in combination regimens were reported to have a favorable response, albeit brief [85,86,87].

## 13. Future Directions

Implementing multi-omic profiling in PBL encounters numerous challenges. At first, sample scarcity—PBL is infrequent, making it challenging to gather large, statistically significant cohorts. Tissue availability is frequently constrained, resulting in small biopsies or stored FFPE specimens, which complicates simultaneous DNA/RNA studies. Secondly, heterogeneity—variations in HIV status, EBV status, and anatomical location—introduces biological variability that interferes with analysis. Third, technical constraints—formalin fixation compromises nucleic acids, rendering high-quality sequencing difficult (although new kits are enhancing this process). Fourth, interpretation—numerous variants of uncertain relevance may be identified, and associating a novel mutation with a therapy warrants research. The clinical evidence levels for targeted medicines in PBL are minimal, resulting in MTB recommendations often being derived from other malignancies. Future research will probably integrate single-cell and spatial multi-omics to analyze tumor and microenvironment heterogeneity in PBL. Single-cell RNA sequencing or ATAC sequencing may reveal subclonal populations or immunological interactions that are overlooked by bulk assays. Spatial transcriptomics/imaging could delineate the niche of EBV+ versus EBV– cells. Proteogenomics, which involves the simultaneous profiling of proteins and RNA, may elucidate post-transcriptional regulators. In the clinic, prospective trials or registries utilizing genomic profiling could evaluate targeted drugs informed by PBL’s mutational profile. Liquid biopsy (cell-free DNA) is a novel instrument that may assist in monitoring minimal residual disease or recurrence in PBL. Ultimately, sophisticated computational techniques, such as machine learning, could enhance the incorporation of intricate multi-omics data into diagnostic classifiers or predictive models. In conclusion, high-throughput multi-omic profiling has significantly enhanced our understanding of PBL’s molecular determinants and has proposed novel diagnostic and treatment approaches.

## 14. Conclusions

Despite the progress made in precision therapeutics and genomic comprehension, the integration of genetic and epigenetic data into standard clinical treatments for PBL remains significantly limited. The low prevalence and variability of PBL make it challenging to establish standardized therapy protocols. Some therapeutic interventions have demonstrated potential; however, others are still under investigation, either experimentally or theoretically.

To precisely identify genetic susceptibilities in various PBL cohorts, future initiatives should focus on expanding genomic and functional investigations. This will facilitate the creation of customized treatment strategies for each patient’s unique characteristics. Furthermore, promoting collaboration between clinical settings and research institutions can enhance the translation of discoveries into effective therapies for PBL. This collaboration can lead to a more streamlined process for validating new therapeutic approaches and ensuring that promising research findings are effectively applied in real-world clinical practice.

## Figures and Tables

**Table 1:** Different clinical features.

Clinical Feature	Plasmablastic Lymphoma	Multiple Myeloma	Large B-Cell Lymphoma
Typical Age Group	Middle-aged to older adults; common in HIV+ patients	Older adults (median ~65–70 years)	Wide age range; more common in older adults
Immune Status	Often immunocompromised (HIV/AIDS, post-transplant)	Generally immunocompetent	Usually immunocompetent
Site of Involvement	Extranodal (oral cavity, GI tract, lymph nodes, skin)	Bone marrow; bone lesions	Lymph nodes; extranodal in 30–40%
Symptoms	Rapidly growing mass, B symptoms, cytopenias	CRAB: hyperCalcemia, Renal dysfunction, Anemia, Bone lesions	B symptoms, lymphadenopathy
Course	Aggressive, rapidly progressive	Chronic but progressive	Variable; indolent or aggressive
Response to Therapy	Poor response to conventional CHOP or EPHOC; high relapse	Responds to new Drugs and Therapies	R-CHOP often effective;
Prognosis	Poor (<1 year in advanced cases)	Variable; improved with modern therapy	Depends on subtype; poor for double/triple-hit
HIV Association	Strongly associated	Not associated	Not typically associated
Diagnostic Challenges	Mimics myeloma; lacks marrow CRAB features	Mimics PBL morphologically; systemic/marrow involvement	Overlaps with PBL in high-grade forms

**Table 2:** Genetic in differential diagnosis of PBL, MM, DLBCL.

	Cell of Origin	Key Genetic Alterations	MYC Dysregulation	EBV Association	Common Mutations	Copy Number Variations	Key Signaling Pathways	Prognostic Markers	Microenvironment
PBL	Post-germinal center B-cell transitioning to plasmablast	MYC rearrangements (50–60%) TP53 mutations (30–50%) PRDM1/BLIMP1 loss	High frequency of MYC translocations (partner: IgL/IgH) MYC overexpression	Strong (60–75% of cases) Linked to HIV/immunosuppression	STAT3 (30%) KRAS/NRAS SOCS1 TET2	Gains: 1q, 8q (MYC locus) Losses: 17p (TP53), 13q	MYC-driven proliferation JAK/STAT activation NF-κB (subset)	MYC rearrangement TP53 loss EBV negativity (worse prognosis)	immunosuppression-driven (HIV, transplant) EBV-mediated immune evasion
MM	Terminally differentiated bone marrow plasma cell	Hyperdiploidy (50%) IgH translocations (t(11;14), t(4;14), t(14;16)) Del(17p) (high-risk)	Rare MYC rearrangements MYC upregulation via secondary mechanisms (e.g., mutations)	Rare	KRAS/NRAS (50%) BRAF (10–15%) TP53 (10%) FAM46C	Gains: 1q21 (CKS1B), 11q Losses: 13q, 17p, 1p	NF-κB (via TRAF3, CYLD mutations) MAPK/ERK (RAS mutations) PI3K	Del(17p) t(4;14), t(14;16) 1q21 amplification	Bone marrow niche (IL-6, VEGF, RANKL) Osteoclast activation
LBCL	Germinal center (GCB subtype) or activated B-cell (ABC subtype)	BCL2 rearrangements (GCB) MYC rearrangements (Double-Hit) BCL6 rearrangements	MYC rearrangements (10–15%) Overexpression in Double-/Triple-Hit lymphomas	Rare (except EBV+ DLBCL in elderly or immune-compromised)	MYD88 L265P (ABC subtype, 30%) CD79B (ABC) EZH2 (GCB) TP53 (20%)	Gains: 3q27 (BCL6), 18q21 (BCL2) Losses: 6q, 9p21 (CDKN2A)	NF-κB (ABC subtype) BCR signaling (MYD88/CD79B mutations) PI3K/AKT	Double-Hit/Triple-Hit (MYC+ BCL2/BCL6) ABC subtype (poor prognosis) TP53 mutations	Dense tumor microenvironment (T-cells, macrophages) PD-L1 expression (subset)

**Table 3:** Epigenetics in differential diagnosis of PBL, MM, DLBCL.

Epigenetic Feature	Plasmablastic Lymphoma	Multiple Myeloma	Large B-Cell Lymphoma
Promoter Methylation	Hypermethylation of PRDM1 and CDKN2A; EBV-driven methylation changes	Global hypomethylation; focal hypermethylation disrupts gene regulation and differentiation	Distinct subtype-specific methylation; TET2 mutations induce hypermethylation
Histone Modifications	Altered by LMP1; changes in H3 methylation; impact on BCR signaling genes	NSD2/EZH2/SMARCA2 interactions alter H3K27me3; EZH2 inhibition induces apoptosis	EZH2 gain-of-function ↑H3K27me3; KMT2D loss ↓H3K4me3 at enhancers
miRNA Dysregulation	miR-150-5p downregulated; associated with E2F3/BIRC5 activation and apoptosis suppression	miR-342-3p, miR10b-5p Hypermethylaion not well highlighted	miR-155, miR150; probably not a central feature in DLBCL epigenetics
Chromatin-Modifying Genes	Mutations in TET2, EP300; affect gene expression linked to JAK-STAT/MAPK pathways	NSD2, EZH2 overactivity maintains oncogenic expression and resistance	Frequent mutations: TET2, EZH2, KMT2D, CREBBP/EP300; impact gene silencing
EBV-Associated Epigenetic Changes	EBV LMP1 affects host chromatin remodeling and promoter silencing	Not a prominent feature	Not EBV-driven in most cases; epigenetic effects may be independent of EBV

arrow up = Hypexpression; arrow down = Hypoexpession
